# Intra-Uterine Medication

**Published:** 1880-11

**Authors:** 


### INTRA-UTERINE MEDICATION.

DESPITE what may be or has been said against the safety of uterine medication, with continued experience and approved methods, we shall certainly continue this plan of meeting certain obstinate conditions of the uterine cavity. At the meeting of the British Medical Association, last August, our distinguished countryman, Dr. Battey, of Georgia, read a paper presenting his mode of intra-uterine medication with a solution of iodine and carbolic acid, to which he has given the name of *iodized phenol.* Dr. Battey's paper is printed in full in the *Virginia Medical Monthly*, current number, and from it we condense. This solution is simply a concentrated solution of iodine in carbolic acid, and Dr. Battey does not consider it at all a chemical compound. In his experiments he prepared an *iodized* phenol of various strengths—one drachm, then two, then three and four drachms of iodine were found soluble in an ounce of glycerine. He says: "The last and strongest solution proved to be decidedly escharotic in its action upon the tissues, and especially upon the heterologous growths of low vitality, and has been used by the writer for attacking uterine cancer—more particular to supplement the curette. The standard solution employed in intra-uterine medication consists of one part by weight of iodine dissolved in four parts of liquified carbolic acid." At first Dr. Battey employed glycerine to dilute the phenol, now he uniformly employs the solution in full strength. Any of the usual forms of applicator wrapped with cotton wool will serve sufficiently well for the application. Dr. Battey uses a slender, elastic, hard-rubber probe, of which he is provided with several ready for use, with the cotton wrapping, for each. We quote now in full his remarks upon his method of application with the results he has observed :

Mode of Application.—The writer selects six or eight of the elastic probes; he then breaks off from the lap of cotton four or five inches of its length, and with his fingers splits it into several fasciculi of such size as when wound upon the probes will enlarge them to a desired thickness. The end of a probe is moistened slightly, and the fasciculus of cotton wound spirally upon it. The cotton around the probe is now dipped into the iodized phenol, any redundancy allowed to drip away, and the probe passed into the uterus with a slow, spiral movement as it advances. At first the probe is introduced but half an inch, the effect noted, and the probe advanced to the internal os if deemed advisable, and then withdrawn. Here, upon a first treatment, the care rests, to note the tolerance of the uterus for the remedy. At subsequent treatments, the probe may be carried to the fundus, and the first probe is followed by a second, and even a third or fourth, if well borne. The remainder of the wrapped probes are employed for wiping off the cervix and vaginal wall, if any of the phenol should have touched these tissues. The energy of the application is regulated by the size of the wrapping, the depth to which the probe is passed, and by the number of probes used. When a very decided impression is to be made, a backward turn is given to the probe in its withdrawal, so as to leave the saturated cotton in the uterus, there to remain twenty-four hours, and often until it is spontaneously expelled. The application is renewed every four or fourteen days, according to the energy of the treatment; in general, once in seven days is sufficient.

The writer has abandoned the use of sponge-tents in connection with the treatment set forth. When dilatation is required, he employs the cotton-wrapped probe, twisting it firmly into the canal by the spiral movement above indicated, and reversing the movement, the probe is withdrawn, and a soft cotton tent remains in the uterine canal. The dilating power of this is notably less than the sponge, but nearly equal to sea-tangle, and it is believed, entirely safe.

The results.—1. A perfect removal of all cervical mucus, which is promptly coagulated, and comes away adhering closely to the cotton. The probes subsequently passed bring the remedy directly in contact with the diseased membrane.

2. Always comparative, and frequently entire freedom from pain. This is a marked feature of the method, and in striking contrast with former experience. Carbolic acid is a local anæsthetic, and so numbs sensibility as to make the energetic application of iodine for the most part devoid of pain.

3. The iodine is so rapidly absorbed by the uterus, that the patient remarks its metallic taste in the mouth and throat usually in five or ten minutes after the application.

4. Softening, and more or less dilatation of the os and cervix.

5. Temporary arrest of leucorrhœa.

6. Watery discharge, sometimes bloody.

7. Exfoliation of the superficial layer of the membrane, which comes away in shreds, and sometimes entire, resembling thin glove kid.

8. Abrasions of the os heal promptly.

9. Disappearance of indurations of the uterus.

10. Permanent arrest of leucorrhœa.

11. Villosities of the endometrium are removed without resort to the curette.

12. Subinvolution of the uterus disappears.

13. The menses become regular and healthy; menorrhagia and scanty menstruation, as well as dysmenorrhœa, are remedied.

14. Appetite and digestion improve, and this in many instances without the use of medicines.

15. So thoroughly is the system impregnated with iodine, alteratives by the stomach are not used.

16. The form of the cervix and os is often completely changed. A large, puffy cervix, with very patulous, slit-like os, becomes even virginal in type.

17. Stenosis has not followed the treatment in any case noted as yet.

18. Barrenness from nine to fourteen years duration has disappeared in several instances.

Remarks.—Rapid, and, at the same time, satisfactory cure in chronic uterine ailments, such as are contemplated in this paper, are not attainable by any mode of treatment known to this writer. It is not proposed that rapid cures can be made by the method herein set forth. On the contrary, the long standing and obstinate cases, such as usually fell into his hands, require many months for satisfactory cure.

